# Leadership and Bullying in the Forestry Organization of Turkey

**DOI:** 10.1155/2017/9454682

**Published:** 2017-09-06

**Authors:** Mahmut M. Bayramoğlu, Devlet Toksoy

**Affiliations:** Faculty of Forestry, Karadeniz Technical University, 61080 Trabzon, Turkey

## Abstract

It is observed that the importance of executive-employee relationships is continuously increasing in today's professional life in addition to the importance of leadership types of managers along with the efficiency of employees as well as the sufficiency of these people in their social relations. Communication of employees with one another and with their manager, along with their social relations, is among the most important factors that sustain an organization. Bullying is a kind of psychological terror that takes place in the form of repeated attacks among workers, as well as by the manager on the employees, aiming to instill stress, job dissatisfaction, and exhaustion on the employees. It has been put forth especially by recent studies that the leadership styles of the managers are highly influential on bullying. The study was carried out with 1189 forest engineers working at 25 different Regional Directorates of Forestry in Turkey. The status of engineers subject to bullying in addition to the effects of leadership types on bullying was determined as a result of the statistical analyses carried out. The results of the study were evaluated comprehensively in comparison with other studies in the relevant literature, thus developing suggestions for preventing bullying behaviors that vary across leadership types.

## 1. Introduction

There has been a significant increase in the number of studies on bullying in the fields of employment and organizational psychology during the past 20 years. Even though this phenomenon is defined in different forms such as workplace aggression, workplace incivility, and emotional abuse in studies carried out in Europe, Asia, and America [1, 2], basically it represents the verbal, psychological, and physical behaviors in professional life that employees or the manager carries out systematically on other employees or the manager, either individually or as groups, which leave the individual desperate and defenseless [3–5]. Employees subject to such behaviors end up with psychological symptoms and psychosomatic and musculoskeletal health problems in addition to emotional reactions such as depression, anxiety, exhaustion, and vulnerability, and they have adverse effects on organizations as well [6–10]. These negativities decrease the performance of the employees in addition to adverse effects like additional costs on the organizations as well as adverse effects on the belief and loyalty of the employees towards their organizations [11]. Even though the number of exclusive studies regarding the costs incurred by organizations as the result of bullying is low, one study revealed that the cost of bullying to organizations in Austria varies between 6 and 36 billion dollars annually [12]. Hence, many studies have been carried out on bullying in organizations in different countries such as Canada [13], Denmark [14], Germany [15], Korea [16], Norway [17], Spain and Belgium [18], Turkey [19], UK [20], and the United States [21]. These studies were generally carried out around a series of topics that concentrated primarily upon the aforementioned adverse effects, whereas studies conducted in our day are built around person-related and work-related factors [22]. The first one is the person-related factors, namely, emotion-focused coping, and it could make employees more vulnerable to bullying [23]. Work-related factors, on the other hand, relate to aspects of the working environment which require sustained physical and/or psychological effort or skills and are therefore associated with certain physiological and/or psychological costs [24]. There are generally more studies on work-related factors in relation to bullying [25]. Examples are role stressors [26], leadership styles [27], and organizational climate [28]. Many researchers have carried out studies in this scope which put forth the relationship of leadership types with workplace bullying and have examined this issue in detail via the generated models [29]. Nyberg et al. [30] also determined a method presenting relationship between workplace bullying and leadership style. In another study, it was found that there was a negative effect of autocratic management style on the workers [31]. In this study, the state of forest engineers working for the government subject to bullying was studied for the first time in detail, aiming at revealing the effects of this phenomenon on the engineers, whereas in the second stage, which comprises the main objective of the study, the types of leadership that the engineers are subject to, functional leadership (FL), paternalistic leadership (PL), transformational leadership (TL), and charismatic leadership (CL) along with their effects on bullying, were examined.

*Types of Leaders and Bullying*. In workplace bullying studies, managers are most frequently indicated as perpetrators by those who are subject to bullying [32]. It has been put forth in these studies that the power and leadership styles of the manager are influential on bullying and theoretical models have been generated by way of this fact which is known as leader bullying behaviors [33–35]. Studies carried out on leadership types and managers have been summarized in Table 1.

When the studies were examined, it was observed that studies had mostly been made on autocratic leadership. The reason for this is generally justified with the fact that any manager generally transforms into autocratic or tyrannical executive type either intentionally or unintentionally as a result of pressure and stress [42]. This leadership style aims at ensuring that the employees in an organization obey the directives of the leader unconditionally. In addition, this has adverse effects especially on the psychological health and performance of the employees in addition to making them feel as if they are treated unjustly and that they are neglected [43, 44].

The study was carried out on functional, paternalistic, transformational, and charismatic leaders. Charismatic leader (CL) theory was first introduced by Weber [45]. In this theory that was later modified by other researchers, CL is defined in terms of the amount of leader influence over followers and as the type of leader-follower relationship [46]. It is put forth in literature that even though this leader type is not required in the organizational culture and strategies of private or public sectors, its adverse effects on organizations and employees are much greater in comparison with its positive effects due to the radical changes it will make [47–50]. Transformational leader (TL) type theory was created by Bass [51] and transformational leadership has been defined primarily in terms of the leader's effect on followers and as the behavior used to achieve this effect. According to this theory, those who follow TL have feelings of trust, awe, loyalty, and respect for their leaders and are motivated above expectations. In addition, this leader type has adverse effects on organizations and employees due to the increased motivation and work load along with high emotional attachment and long-term stress [52, 53]. McGrath [54] introduced functional leadership (FL) theory and stated that it is very important in teamwork, especially for determining the roles of team leaders in detail. This method, which generally has a positive effect on increasing organizational efficiencies [55], has adverse effects on employees such as excessive work load and stress. Paternalistic leadership (PL) has been defined as “a style that combines strong discipline and authority with fatherly benevolence” [56] in East Asia, with three distinct dimensions: authoritarianism, benevolence, and morality. PL is also the prevalent leadership style in business organizations of the southeastern countries. Of the three dimensions, while benevolence and morality have positive effects on employees, authoritarianism has negative effects on employees [57].

**Figure 1:**
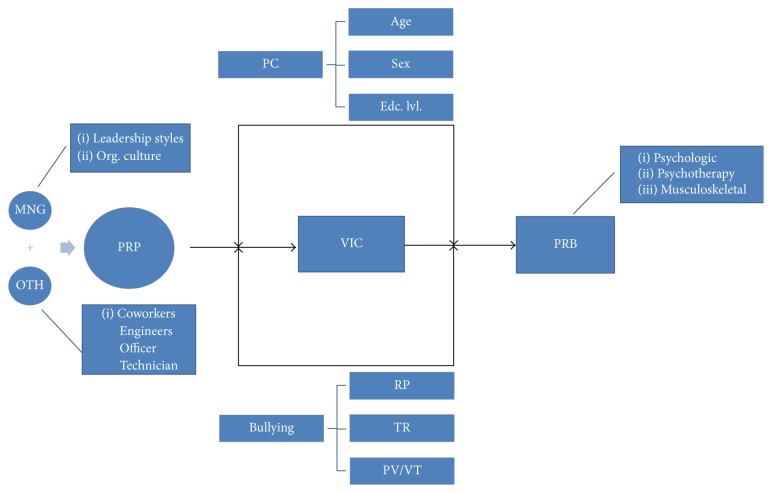
Concept of workplace bullying.

*Conceptual Framework*. Workplace bullying studies are generally carried out on behaviors that result in adverse effects on people or the organization. There are studies on the relationship between the leadership styles in organizations and this concept. However, the number of studies on leadership types-bullying, which are summarized in Table 1, is limited. In addition to these studies, those that have been carried out on workplace bullying in Turkey, especially in the government sector [58–65], were examined, and the concept in Figure 1 was generated.

Even though managers (MNGs) come into prominence as perpetrators more often in workplace bullying, the coworkers (OTH) of the victims (VICs) also appear as PRP. Soylu [66] stated that individuals who are at managerial positions in the government sector are less subject to bullying in comparison with those who are not and pointed out MNGs as PRP, whereas Vartia and Hyyti [67] determined that VICs are also being bullied by coworkers besides the MNGs. In this respect, the concept of PRP was divided into two as MNG and OTH. Engineers, clerks, and technicians working at the institution were evaluated as OTH within the scope of the study. The leadership types of MNGs as well as the culture and organizational structure of the institution are all effective on bullying [68]. As an example, forest engineering in Turkey is a male-dominated occupational group. While 36% of the women at male-dominated businesses in America see themselves as bullying victims, this ratio is 5% in Norway [69]. In Turkey, on the other hand, this ratio is 23% [23]. Hence, the organizational structure was also taken into consideration in addition to the leadership types of MNGs. Bullying is a less well recognized issue in developing countries, including Turkey [70]. That is why the effects of personal characteristics (PC) on bullying and health problems afterwards (PRB) are greater in comparison with developed countries. Even though bullying behaviors are classified under different numbers and names due to cultural differences of countries, their levels of development, masculine/feminine values, and the methods used in the studies [71], their effects are observed in the way the victims face such behavior as well as the PRB they face afterwards.

## 2. Methodology

The study consists of a questionnaire implemented through face-to-face interview with forest engineers in Regional Directorates of Forestry (RDF) in Turkey between 2013 and 2014. The research comprises three chapters and 33 questions. The first part of survey treats demographic information, the second chapter concentrates on psychological harassment behaviors and frequency of exposure, and the last section focuses on how often employees come across such behaviors in work environment and who exhibits such behaviors. The study uses Behavioural Experience Method developed by Einarsen and Skogstad [3] which consists of 22 questions in consideration of characteristics of the participant group. The method enables determining whether participants came across bullying in the last 6 months. In addition to that, Revised Version of the Negative Acts Questionnaire (NAQ-R) with a Likert scale of 5–7 points was employed so as to identify exposure frequency and amount and the agent of bullying. The frequency of exposure to psychological harassment in the last 6 months was expressed with the aid of NAQ-R via options “never, very rare, at least a few times every month, at least a few times every week, and at least a few times every day.” The frequency of presence of bullying in the institution was expressed on a 5-point Likert scale, namely, “every day, every week, every month, rarely, and never.” Survey data were assessed by means of frequency and crosstabs, as well as factor analysis, *t*-test, and ANOVA, which enabled formation of principal factor groups so as to understand and interpret the relation between questions.

A questionnaire consisting of 32 questions was applied to the participants following the bullying questionnaire in order to determine the manager types of those who participated in the questionnaire. Functional Team Leadership Scale (FTLS) in Santos et al. [72] was used for FL while preparing these questions, Cheng et al. [73] was used for PL, Multifactor Leadership Questionnaire (MLQ) was used in Bass and Avolio [74] for TL, and C-K Scale in Conger and Kanungo [75] was used for CL.

Leadership types were determined with a 5-point Likert scale and Multinomial Logistic Regression (MLR) was used to determine the effect of leadership type on the factor groups.

## 3. Results

The survey was conducted with forest engineers working at 25 RDFs operating under Ministry of Forestry and Water Management. A total of 1253 questionnaires were implemented; 64 were excluded from assessment due to lack of data; thereupon, the analyses and assessments were performed on 1189 questionnaires. Face-to-face interview method was used in the survey. 75.6% of participants are male, 76.3% are married, and 44.7% are between the ages of 33 and 44. Among the participating forest engineers, 21.3% are postgraduates, while 26.3% work in the organization for 1 to 5 years. 19.2% of participants are employed in administrative positions in the institution. Forest engineers have served in an average of 2 units within the institution in the last 10 years. Demographic particulars of participants are given in Table 2.

The intention was to form principal factor groups so as to facilitate comprehension and interpretation of the relation between the questions in the survey. For this purpose, factor analysis was run on 22 of the questions. Suitability of data for factor analysis is tested by means of Kaiser-Meyer-Olkin (KMO) coefficient and Bartlett's sphericity test. KMO coefficient provides information on whether data matrix is suitable for factor analysis. KMO should be higher than .60 for factorability. In the present study, the mentioned value was calculated as 0.948. Moreover, Bartlett's sphericity test was examined (*χ*^2^: 10232.051; *p* = 0.000 < 0.01); the resulting data proved convenient for factor analysis. The factor structure of this instrument was analyzed using principal component analysis with varimax rotation. At the end of the analysis, 3 factors with eigenvalue of more than 1.00 which explain 51.4% of total variance were determined. Screen plot was also examined and it was concluded to evaluate NAQ-R under 3 factor groups. The first factor group is relevant to person (RP) concerning the 11 questions about personality. The second one is tasks related (TR) concerning 6 questions about vocation of forest engineers. The last one is physical violence/verbal threat (PV/VT) related to 5 questions about physical attacks or verbal threats at workplace. The factor structure and loadings are given in Table 3. In the study, variables with factor loadings ≥ .40 were selected for inclusion to maximize factor interpretability. Cronbach's *α* value of NAQ-R was found to be 0.921 for the research.

As can be seen in Table 3, Cronbach's alpha values of RP, TR, and PV/VT are 0.88, 0.79, and 0.70, respectively. 52.8% of participants are subject to such behaviors of their fellow engineers, albeit rarely; 52.9% witness other colleagues suffer from mentioned behaviors. ANOVA and *t*-test were put to use in order to reveal relation between demographic traits of participants and the above-given factor groups. Moreover, crosstabs and frequency tables were also used. The *t*-test results are shown in Table 4.

When the analysis results were examined, a statistically significant relationship was determined between gender and only tasks related, *t*_(2.662)_ = 0.008, since *p* ≤ 0.05. Accordingly, female forest engineers who participated in the questionnaire (19.4%) put forth that they were subject to tasks related behavior more often in comparison with their male colleagues (17.4%). Of the female engineers, 47.5% are subject to carrying out works that are below their experience, skill, and education levels, whereas 32.3% of the males are subject to excessive workload that they cannot cope with. No relationship was determined between the position inside the institution and the factor groups as a result of the analysis.

Results related to the determination of the relationship between age, marital status, education level, and service time and factor groups are presented in Table 5.

It was determined as a result of the test that there was a statistically significant relationship between the ages of the forest engineers who participated in the questionnaire and relevant to person (*F*_(3.136)_ = 0.014, *p* ≤ 0.05) and tasks related (*F*_(9.245)_ = 0.000, *p* ≤ 0.05) behaviors. According to these results, those who are in the 2nd age group are subject to relevant to person and tasks related behaviors the most. When RP is examined according to age groups, it was determined that engineers in the 2nd, 3rd, and 4th age groups believe that information they think that will affect their success is hidden from them at ratios of 19.5%, 14.3%, and 33.3%, respectively. Moreover, 21% of the young engineers who just started their jobs think that the most unimportant and undesired tasks are assigned to them. When age and TR were examined, it was found out that the engineers in the 1st and 2nd age groups believe at ratios of 31.4% and 31.1%, respectively, that they are subject to workload they cannot cope with, whereas 46.4% of those in the 3rd age group think that unimportant and undesired tasks are assigned to them. It was determined that engineers in the 4th age group were not subject to any bullying behavior regarding workload. It was determined that young engineers (between the ages of 23 and 33) are subject to bullying more often than their elderly colleagues. No relationship was determined between the marital status of the engineers and the ratio with which they are subject to bullying. There was a statistically significant relationship between the level of education and only physical violence/verbal threat (*F*_(4.283)_ = 0.005, *p* ≤ 0.05). As the level of education increases, 49.2% of the engineers are subject to carrying out works that are below their experience, skills, and education levels. That is, forest engineers with a doctorate degree are subject to more physical violence/verbal threat behaviors in comparison with their colleagues who have bachelor's or master's degrees. A relevant to person (*F*_(3.016)_ = 0.010, *p* ≤ 0.05) and tasks related (*F*_(5.838)_ = 0.000, *p* ≤ 0.05) relationship was determined between the service times of the forest engineers who responded to the questionnaire and the levels at which they are subject to bullying. It was determined that as the experience at work increases, ideas and opinions of 46.3% of the engineers are not taken into consideration, and 41.2% are subject to carrying out works below their experience and level of education.

The study examined the health problems faced by engineers in case of exposure to this kind of behavior. A total of 1461 records were obtained. Exposure to bullying in the workplace caused dispiritedness (37.9%), insomnia (13.8%), headache (12.5%), feeling of quitting the job (9.8%), and stomachache (9.2%). The health problems that forest engineers observed or witnessed in coworkers were investigated. A total of 1448 records were obtained under this topic. 43% of the respondents indicated that their friends were exposed to these behaviors. They noted that their friends who were exposed to these behaviors met with health problems such as quitting the job (13%), headache (9.6%), insomnia (8.4%), and stomachache (7.8%).

Leadership types were determined according to the results of the 5-point Likert scale. Engineers define their managers as TL ($\overset{-}{x}$ = 3.54). The classification after TL is as follows: FL ($\overset{-}{x}$ = 3.49), PL ($\overset{-}{x}$ = 3.41), and CL ($\overset{-}{x}$ = 3.33). Multinomial Logistic Regression (MLR) was applied to determine the effect of the leadership type on the factor groups (RP, TR, and PV/VT) obtained in the study (Table 6).

The model generated as a result of MLR (−2LL = 1238.89; *X*^2^ = 271.618; df = 189; and *p* = 0.000 < 0.05) was statistically significant. When the analysis results regarding the effects of leadership types on bullying behavior were examined, it was observed that the RP (*p* = 0.009 < 0.05) and TR (*p* = 0.000 < 0.05) behaviors that engineers working at the institution are subject to vary depending on the type of their leaders. Accordingly, when TR behaviors were examined, it was determined that engineers with functional and charismatic managers are most frequently subject to workloads they cannot cope with and those with paternalistic manager types are subject to unjust criticism, whereas those with transformative leader as their manager are forced to carry out works that are below their level of experience and skills. When RP was examined, it was determined that engineers with functional and transformative leaders feel uncomfortable about information that might affect their success being kept away from them and those with paternalistic manager types are uncomfortable about their opinions and ideas not being taken into consideration, whereas those with a charismatic leader as their manager feel uncomfortable about unfounded gossips related with them.

## 4. Discussion

The total variance explained by the 3-factor solution obtained as a result of the factor analysis is at a sufficient level for the studies in social sciences [76]. Factor group structures obtained via factor analysis bear similarities with those in Einarsen and Raknes [77], Baron et al. [78], and Galanaki and Papalexandris [79]. In the study, the existence of a statistical cause-effect relationship between the personal characteristics of the bullying victims and their states of being subject to such behavior was examined in the concept generated via ANOVA and *t*-test. In this scope, the relationship between gender and bullying was determined similar to that of Trijueque and Gomez [80] and Moreno-Jimenez et al. [81]. It was revealed in the study that females are subject to bullying more in comparison with their male colleagues as is the case in male-dominated occupations as put forth similarly by Salin [82] and Lewis and Gunn [83]. It was determined in the study that there is a relationship between age and bullying in parallel with the results of the study by Rayner and Hoel [84]. Similar to the studies by Aydan et al. [85], young forest engineers are subject to such behavior more in comparison with their elder counterparts. The reason for this is the quality and quantity of the tasks assigned to young engineers due to their lack of experience. The bullying behaviors of engineers vary according to their levels of education as was displayed by Manotas [86]. Similar to Ertürk and Cemaloğlu [87], engineers who have completed their doctorate programs are subject to more bullying compared to their other colleagues. Toksoy and Bayramoğlu [23] explain the reason for this as follows: bullying perception increases in correlation with the level education due to the fact that individuals with higher education levels have more developed abilities to work independently, think, and decide on behalf of their managers and that they have greater sense of managerial and personal responsibility along with criticism skills, thus leading to jealousy among their colleagues. A statistically significant relationship was determined between the number of years the engineers have been working at the same institution and bullying similar to the study by Üye [88]. Being subject to bullying behavior increases in parallel with the number of working years, as is the case in Atasoy [89]. It is inevitable that incompetent people are assigned to senior positions in Turkey and that they have a difficult time of establishing authority in the institution, since they are assigned to these positions mostly by way of political maneuvers. This also has a negative impact on the relationships between the managers and those who are managed. It is observed that such managers are insensitive towards the problems of their own personnel and that they force their employees to carry out their political demands [60]. Despite the fact that the leadership types of managers play an important role in minimizing the aforementioned negativities, they may also be a potential cause of bullying observed in organizations [90]. It was found out in the study that leadership types are influential on bullying. Transformative leadership explains not the leadership effect on a group or on organizational processes but the direct effect on individual followers. Similar to the findings of Porter and Bigley [91], it was determined that forest engineers with transformative leaders are subject to negative behaviors that will affect their success and work quality as a result of the competition in the institution. On the other hand, engineers with charismatic leaders are subject to limited access to knowledge, which might be effective on the performance of engineers, as is the case in Yukl [46]. Chen et al. [57] stated that paternalistic leaders instill feelings of fear and anger on their employees. In the study, this emotion was determined as anger in the forest engineers with paternalistic leaders. The reason for this is the unjust criticisms that they are subject to in addition to their not being taken into consideration. In parallel with the findings of Dechurch and Marks [55], forest engineers with functional leaders are subject to excessive overload. Since this study has a cross-sectional design, the correlations obtained from the study could be reversed according to physical, mental, and economic conditions of the respondents. In addition, although the correlation is not possible to determine the time alignment of the relationship, it is important not to give definite causality but to reveal the influence and frequency of leadership types on bullying in terms of public efficiency.

## 5. Conclusion

In the study, the opinions of forest engineers on bullying and their status of being subject to bullying according to their demographic properties were examined. Statistical analyses were carried out on data acquired via questionnaires implemented for this purpose. Data that support those of the similar studies in relevant literature were acquired as a result of the analyses. Bullying behavior may change with respect to geographies and cultures. These behaviors are not approved in Turkey and majority of the victims consider this as a source of stress and an embarrassing situation. The fact that engineers are employed by the government and that they hide their exposure to bullying has resulted in the bullying ratios to be lower than expected in comparison with other studies. According to the results obtained from this study carried out in the forestry organization, knowledge and awareness regarding the subject of bullying are on the increase. The number of studies on this issue has to be grown in order to reveal bullying in the forestry organization in a more detailed manner and to analyze it. This study is valid only for the forestry sector and further studies that take into consideration the organizational structure, type, working environment, legal status, and so forth should be carried out in order to reach a definite judgment.

Management brings along many responsibilities. People at management levels can meet these responsibilities only with their knowledge, skills, and experience. Hence, an egalitarian approach should be used especially in selecting managers, internal promotions, and appointments; due importance should be given to merit. Negative effects of managers on the employees result in the organization facing many different financial and emotional problems, regardless of the type of management. That is why managers should be effective and have a just management understanding. Managers should take into consideration the knowledge, skill, and abilities of their subordinates in addition to their emotions and thoughts, trying to instill a sense of corporate belonging in each of them.

## Figures and Tables

**Table 1:** Literature about leadership types and manager.

Authors	Paper Design	Stressors
Agervold and Mikkelsen [36]	The Psychosocial Work Environment and Stress Questionnaire (PWSQ)	Autocratic management style
	Negative Acts Questionnaire (NAQ)	
	*t*-test and chi-square	
	*N* = 186	

Hauge et al. [7]	NAQ-Revised	Tyrannical leadership; laissez-faire leadership
	MANOVA	
	*N* = 2539	

Nyberg et al. [37]	Multiple logistic regression analyses	Inspirational leadership, autocratic leadership, and self-centered leadership
	*N* = 5141	

Hoel et al. [38]	Structural equation models	Autocratic leadership; participative leadership; noncontingent punishment (NCP leadership); laissez-faire style of leadership
	*N* = 5288	

Westerlund et al. [39]	Logistic regressions	Attentive managerial leadership
	*N* = 12622	

Oxenstierna et al. [40]	Multiple logistic regressions	Lack of trust in leadership, dictatorial leadership, bad relationship to closest superior
	*t*-test	
	*N* = 2203	

Mihalcea [41]	*t*-test; chi-square	Laissez-faire Transactional leadership
	*N* = 1272	
	The Multifactor Leadership Questionnaire (MLQ)	

**Table 2:** Demographic characteristics of forest engineer.

Characteristics of participants	Percent (%)	*N* (person)
Gender		
Male	75.6	899
Female	24.4	290
Age groups		
23–33	31	368
34–44	44.7	532
45–56	20.3	241
56+	4	48
Education level		
Bachelor's	78.7	936
Master's	19.3	230
Doctorate	2	23
Marital status		
Married	76.3	906
Single	21.2	253
Widow/divorced	2.5	30
Work years		
1–5	26.3	312
6–10	24.2	288
11–15	16.3	194
16–20	14.6	175
21+	18.5	220

*Total*		*1189*

**Table 3:** Factor analysis for bullying instrument.

Kaiser-Meyer-Olkin (KMO)	.948	Relevant to person (RP)	Tasks related (TR)	Physical violence/verbal threat (PV/VT)
Approx. Chi-Square	10232.051			
df	231			
Sig.	*p* (0.000) < 0.005			
Bartlett's sphericity	*X* ^2^: 10232.051; *p* = 0.000 < 0.01			
Cronbach's *α* value of NAQ-R	0.921			
Factor loadings		≥.40	≥.40	≥.40
Cronbach's alpha		0.88	0.79	0.70
Q1. Did anyone at your workplace hide information from you which you believe would affect your success?		0.618		
Q2. Have you ever been insulted or humiliated regarding your performance?		0.613		
Q4. Have you ever been assigned insignificant, unwanted and undesirable tasks other than those which are your own responsibility and your task?		0.672		
Q5. Did anyone ever spread an unfounded rumor about you, or gossip about you?		0.578		
Q6. Have you ever felt that you were ignored or excluded, or that you were treated as if you were worthless?		0.758		
Q7. Have you ever been insulted regarding your personality, your attitudes, your private life or your values?		0.664		
Q8. Have you ever been exposed to unfounded sudden anger or fury at the workplace? Have you ever been yelled at for no reason?		0.516		
Q10. Has it ever been implied that you should quit the job?		0.589		
Q12. Have you ever been ignored, neglected or mistreated by your co-workers?		0.687		
Q13. Were your work activities or projects subjected to unfounded criticism?		0.483		
Q14. Have you ever felt that your ideas and opinions were neglected?		0.542		
Q3. Have you ever been forced to perform duties beneath your experience, capacity and education level? Were you ever asked to do such work?			0.533	
Q11. Have you ever been reminded of your previous mistakes in respect of the job?			0.472	
Q16. Have you ever been asked to perform unreasonable or time-limited tasks that are impossible to complete?			0.814	
Q18. Have you ever been subjected to excessive supervision beyond the normal standards?			0.583	
Q19. Have you ever had a heavy workload that you could not manage?			0.522	
Q21. Have you ever been forced into not claiming your legal rights (annual leave, sick leave, travelling expenses etc.)?			0.824	
Q9. Did anyone ever make a threatening gesture towards you? Or have you ever been pushed, physically blocked or exposed to other such physical behaviors?				0.538
Q15. Have you ever been exposed to undesired “fun and games” by people you have problem with?				0.435
Q17. Have you ever experienced serious denunciation, accusations or incrimination?				0.526
Q20. Have you ever been exposed to derisive conversations, verbal abuse or sarcasm?				0.481
Q22. Have you ever experienced ill-treatment or physical or sexual harassment?				0.724

**Table 4:** The relationship between the demographic characteristics and factor groups according to *t*-test.

Variables	Component								
	Relevant to person			Tasks related			Physical violence/verbal threat		
	df	*t*	Sig.	df	*t*	Sig.	df	*t*	Sig.
Gender	1129	1.621	0.105	1129	2.662	**0.008** ^**∗**^	1129	1.170	0.242
In-house position	1123	1.825	0.068	1123	0.908	0.364	1123	1.522	0.128

^*∗*^ *P* < 0.05.

**Table 5:** The relationship between the demographic characteristics and factor groups according to ANOVA.

Variables	Component								
	Relevant to person			Tasks related			Physical violence/verbal threat		
	*F*	df	Sig.	*F*	df	Sig.	*F*	df	Sig.
Age	3.136	1132	**0.014** ^**∗**^	9.245	1132	**0.000** ^**∗**^	0.409	1332	0.802
Marital status	1.206	1133	0.300	1.066	1133	0.345	1.509	1133	0.221
Education level	1.234	1133	0.296	0.688	1133	0.560	4.283	1133	**0.005** ^**∗**^
Duration of the professional life	3.016	1134	**0.010** ^**∗**^	5.838	1134	**0.000** ^**∗**^	1.875	1134	0.096

^*∗*^ *P* < 0.05.

**Table 6:** The effect of the leadership type on the factor groups according to MLR.

Factor groups	−2 log likelihood of reduced model (−2LL)	Chi-square (*χ*^2^)	df	Sig.
RP	1360.394	121.497	87	**0.009** ^**∗**^
TR	1343.749	104.853	57	**0.000** ^**∗**^
PV/VT	1285.468	46.571	36	0.112

^*∗*^ *P* < 0.05.
